# Protein nutritional support in critically ill patients: pathophysiological basis, clinical evidence, and areas of uncertainty – a narrative review

**DOI:** 10.3389/fmed.2026.1770345

**Published:** 2026-04-10

**Authors:** Du Qinyuan, Qin Congcong, Li Guochen, Bi Qianyu, Batejin, Zhang Shuanglin, Bao Bagena, Bao Meiyu, Zhang Yimin, Sun Meiling, Zhang Sichao, Li Kong

**Affiliations:** 1 First Clinical Medical College, Shandong University of Traditional Chinese Medicine, Jinan, Shandong, China; 2 Institute of Traditional Chinese Medicine Literature and Culture, Shandong University of Traditional Chinese Medicine, Jinan, Shandong, China; 3 International Mongolian Hospital of Inner Mongolia Autonomous Region, Hohhot, Inner Mongolia, China; 4 School of Traditional Chinese Medicine, Shandong University of Traditional Chinese Medicine, Jinan, Shandong, China

**Keywords:** burns, critical illness, medical nutrition therapy, protein supplementation, sepsis

## Abstract

Protein metabolic derangement is a hallmark of critical illness and contributes to adverse outcomes and delayed recovery. This narrative review synthesises current evidence on protein metabolism, requirement assessment, and supplementation strategies in critically ill adults, with emphasis on sepsis and severe burns as representative hypercatabolic phenotypes. Sepsis and major burns are characterised by accelerated skeletal muscle breakdown, negative nitrogen balance, and sustained functional impairment. Current guidelines generally support progressive enteral-first protein delivery after haemodynamic stabilisation, targeting at least 1.2–1.3 g/kg/day, whereas higher targets are more consistently justified in severe burns because of prolonged hypermetabolism, exudative losses, and wound-healing demands. However, randomised trials and meta-analyses do not show a uniform mortality benefit from higher protein provision, and very high early doses may be harmful in selected patients, particularly those with acute kidney injury or poor metabolic tolerance. Evidence for specific protein sources, functional amino acids, micronutrients, probiotics, and metabolic modulators remains heterogeneous. Future research should define phenotype-specific protein strategies using metabolic, structural, functional, and long-term patient-centred outcomes.

## 1 Introduction

Among these, disordered protein metabolism and negative nitrogen balance are closely linked to muscle wasting, delayed wound healing, increased risk of infection, prolonged mechanical ventilation, and impaired rehabilitation (1–4). These associations have highlighted protein metabolism as a key determinant of both short-term mortality and long-term functional outcomes and have shifted protein intake from a secondary component of energy provision to a central therapeutic target in nutritional support for critically ill patients (1, 2). However, current guidelines and clinical trials yield inconsistent conclusions regarding the optimal dose, safety upper limit, timing of initiation, and route of protein administration in this population (2, 4). Substantial uncertainty also remains concerning the true impact of different protein sources (such as whey, casein, and plant-based proteins), functional amino acids, and related modulatory factors on protein metabolism and clinical outcomes (1, 4). On this basis, we use sepsis and severe burns as representative conditions to synthesise basic and clinical evidence on protein metabolism and supplementation in critical illness, with particular emphasis on unresolved issues related to dose, timing, protein sources and modulatory factors, and to outline pragmatic directions for clinical practice and future research. This narrative review addresses a pragmatic clinical question: how should protein supplementation be operationalised across phases of critical illness—particularly in sepsis and severe burns—with respect to timing, dose range, risk gating, and areas of evidence uncertainty. Rather than reiterating guideline statements, we use sepsis and severe burns as two hypercatabolic phenotypes to examine where evidence converges, where it conflicts, and where it remains insufficient for bedside decision-making. Throughout, we prioritise phase-adapted implementation, clinically actionable constraints (tolerance and safety), and outcomes beyond short-term mortality, including organ dysfunction, wound healing, and functional recovery.

### 1.1 Literature acquisition and selection

To increase methodological transparency, we conducted a targeted literature search in PubMed/MEDLINE covering the period from 1 January 2000 to 31 October 2025; the final search was performed on 31 October 2025. Search terms were organised into three concept blocks and combined with Boolean operators: (i) setting/population (critical illness, intensive care, ICU), (ii) nutrition/protein exposure (protein intake, high-protein, protein supplementation, amino acids, enteral nutrition, parenteral nutrition), and (iii) phenotype (sepsis, septic shock, burn injury). Controlled vocabulary and free-text terms were used where appropriate. Eligibility was defined pragmatically as adult ICU populations, studies addressing protein dose, timing, route, or specific protein/amino-acid products, and reporting at least one clinically relevant endpoint and/or quantitative index of protein metabolism. The focus on sepsis and severe burns reflects their shared hypercatabolic physiology, high risk of lean-tissue loss, and persistent uncertainty regarding phase-adapted protein targets and safety trade-offs.

## 2 Protein metabolism in critically ill patients: sepsis and severe burns

### 2.1 Alterations in protein metabolism in sepsis and severe burns

In critical illness, whole-body metabolism shifts from anabolism toward predominant catabolism, with skeletal muscle protein becoming the major nitrogen source under stress conditions. Both trauma and sepsis markedly increase whole-body protein breakdown, with elevated rates of muscle protein degradation and relatively insufficient synthesis, leading to the rapid development of negative nitrogen balance that is closely associated with ICU-acquired weakness and long-term functional impairment (5–9). During this process, large quantities of essential amino acids are released from skeletal muscle and utilised for the synthesis of acute phase proteins, immune effector molecules, and reparative tissues, resulting in a persistent depletion of whole-body protein stores.

In sepsis, systemic infection and the associated inflammatory cytokine storm activate a complex neuroendocrine network, leading to increased levels of glucocorticoids, catecholamines, and glucagon. These hormones accelerate skeletal muscle proteolysis while enhancing hepatic acute phase protein synthesis, resulting in net nitrogen transfer from muscle to liver (9, 10). Experimental studies indicate that sepsis-associated muscle atrophy is mainly attributable to enhanced protein degradation, whereas the reduction in protein synthesis is relatively less pronounced (5, 6, 8). Amino acid metabolism is also substantially altered: some studies have reported decreased plasma concentrations of most amino acids and accelerated transcapillary transport of amino acids into tissues, suggesting a functional deficiency under high catabolic load (10–12). Other studies have found that when severe catabolism coexists with impaired hepatic synthetic capacity, circulating levels of certain amino acids increase but cannot be effectively converted into skeletal muscle or other functional proteins, reflecting reduced amino acid utilisation efficiency and a blunted protein synthetic response (10–12). Clinically, this metabolic pattern, characterized by rapid loss of muscle protein, reduction in lean body mass, and redistribution of protein stores, represents an important pathophysiological basis for increased protein requirements and difficulty in long-term muscle strength recovery in patients with sepsis (5–8, 10, 11).

Severe burns represent a prototypical condition of systemic hypermetabolism and exaggerated protein catabolism after injury. Following extensive burns, persistent activation of the sympathetic nervous system and prolonged elevation of stress hormones can increase resting energy expenditure to approximately 1.5–2.0 times normal values, accompanied by marked skeletal muscle protein breakdown and loss of lean body mass (3, 13–15). Relevant studies have shown that patients with severe burns may remain in a hypermetabolic and hypercatabolic state for months after injury, with body weight decreasing by approximately 20–25%. Inadequate protein supplementation under these conditions can significantly delay wound healing and increase the risk of infection and organ dysfunction (3, 13–15). Similar to sepsis, patients with burns also exhibit altered patterns of amino acid utilisation, but with distinctive features: additional protein and micronutrient loss due to wound exudation and wound protein synthesis is more pronounced, and the hypermetabolic state is more prolonged, such that short-term intensive supplementation is insufficient to fully offset the long-term negative nitrogen load (3, 13–15).

Taken together, current evidence indicates that sepsis and severe burns share common features of protein metabolic derangement, including markedly enhanced skeletal muscle proteolysis, rapid loss of lean body mass, preferential allocation of amino acids to acute phase responses and tissue repair, and substantial depletion of whole-body protein reserves (5–8, 11, 13–15). At the same time, important differences exist between the two conditions. Patients with severe burns exhibit a higher degree and longer duration of hypermetabolism and additional exudative protein losses from burn wounds, which increase their dependence on exogenous protein supply compared with most patients with sepsis (3, 13–15). These differences imply that a single, uniform strategy for protein supplementation in terms of dose, duration, and evaluation methods is unlikely to be suitable for both populations.

#### 2.1.1 Controversy and gaps

It remains uncertain whether higher protein provision can meaningfully overcome early-phase anabolic resistance and improve patient-centred outcomes beyond biochemical surrogates, given heterogeneity in illness severity, immobility, and concurrent organ support that may modify utilisation and tolerance. Future studies should model phase–phenotype interactions and prioritise functional recovery endpoints alongside short-term outcomes.

### 2.2 Assessment and monitoring of protein requirements

In critically ill populations, such as those with sepsis and severe burns, accurate assessment and dynamic monitoring of protein requirements are essential for developing individualised supplementation strategies. Empirical formulae based on actual or ideal body weight do not adequately reflect true needs under stress conditions, particularly in the setting of large-volume fluid resuscitation, generalised oedema, and shifts in tissue fluid, where determining protein doses solely according to body weight can lead to substantial misestimation (2, 16–18). Current guidelines therefore provide recommended ranges as practical starting points: the ESPEN guidelines suggest that protein intake in critically ill patients should gradually reach approximately 1.3 g/kg/day during the middle phase of illness (2), while several societies and expert consensus documents recommend a range of 1.2–2.0 g/kg/day, with adjustment according to clinical phenotypes such as age, obesity, trauma or burns, and acute kidney injury (16–18). These values offer a safety range and directional reference for clinical practice but do not replace individualised assessment.

A combination of metabolic and structural indicators is advisable for monitoring protein adequacy. Nitrogen balance estimation remains a classic method for judging whether protein intake approximately meets requirements (18). By quantifying protein intake and measuring 24-h urinary urea nitrogen excretion, net nitrogen balance can be used to determine whether the patient is in pronounced negative nitrogen balance, near equilibrium, or mild positive nitrogen balance, although this approach is influenced by the completeness of urine collection and assumptions regarding non-urinary nitrogen losses and is therefore more suitable for trend monitoring than as an absolute decision criterion (18). In addition, assessment of body composition and skeletal muscle mass has become increasingly important. Bedside muscle ultrasound, together with indices such as prealbumin, may facilitate dynamic evaluation of lean body mass changes and response to nutritional intervention (6, 19, 20), whereas computed tomography and dual-energy X-ray absorptiometry can quantify lean body mass more precisely, albeit with limited ICU feasibility (19). Traditional biochemical markers such as albumin, prealbumin, and transferrin mainly reflect inflammation and fluid status in the acute phase and should not be used as surrogate markers of total body protein or short-term response to nutritional therapy (20, 21).

From the perspective of disease phenotypes, assessment of protein requirements in sepsis should account not only for heightened catabolism but also for haemodynamic stability, renal function, and inflammatory burden. Rapidly increasing protein doses during phases of highly active inflammation and severe organ dysfunction may not confer net benefit and may even be associated with adverse outcomes in a subset of patients with concurrent acute kidney injury (22, 23). In contrast, patients with severe burns often require higher protein delivery because of prolonged hypermetabolism, exudative protein losses, and ongoing wound repair, with some studies and guidelines suggesting that protein intake in relatively stable phases may need to reach or exceed 1.5–2.0 g/kg/day (24, 25). Overall, assessment and monitoring of protein requirements in critically ill patients should move from fixed-formula approaches toward dynamic strategies that integrate guideline ranges, nitrogen balance, body composition monitoring, organ function, and disease phenotype (2, 22, 24, 25).

## 3 Principles of protein supplementation

### 3.1 Physiological mechanisms of protein supplementation

In critical conditions such as sepsis and severe burns, the organism transitions from homeostasis to a stress-related metabolic pattern dominated by catabolism, with skeletal muscle protein being extensively mobilised to provide nitrogen and carbon skeletons for the synthesis of acute phase proteins, immune responses, and tissue repair (6, 9). Against this background, the core objective of exogenous protein supplementation is to provide an adequate substrate with an appropriate amino acid profile to maintain skeletal muscle and visceral protein pools, support immune cell function, and promote restoration of organ structure and function (17, 26).

Functional amino acids, including glutamine and arginine, have biological plausibility in critical illness, but their clinical benefit and safety are likely dependent on phenotype and illness phase, and evidence-based recommendations are addressed in Sections 6.4 and 6.5 (27–29). Therefore, they should be considered within an illness-phase and tolerance-guided framework rather than as routine adjuncts to protein delivery (9, 27–30).

At the level of muscle and organs, critically ill patients typically exhibit substantially higher rates of muscle protein breakdown than synthesis, resulting in rapid loss of lean body mass and ICU-acquired weakness, which directly affect weaning from mechanical ventilation, functional mobility, and long-term outcomes (6, 9). Proteins rich in leucine can enhance muscle protein synthesis via activation of the mTOR pathway and thereby partially overcome anabolic resistance in critical illness, attenuating negative nitrogen balance (30, 31). The liver increases acute phase protein synthesis under stress and thus has significantly increased demands for protein and amino acids, whereas the kidney contributes to whole-body nitrogen metabolism through amino acid deamination and acid–base regulation (9, 26). Consequently, rational protein supplementation not only helps preserve skeletal muscle but also provides substrate support for the recovery of hepatic, renal, and other vital organ functions (6, 9, 26).

Recent studies have suggested that in certain critical illness phenotypes, such as those with concomitant renal dysfunction or very high early inflammatory burden, simply increasing protein intake does not necessarily translate into improved functional outcomes and may even be associated with higher rates of adverse events (22, 23). These findings indicate that protein supplementation strategies should be aligned with the phase of illness, organ function, and metabolic tolerance, rather than focusing solely on dose escalation as the primary goal.

### 3.2 Protein types and absorption

At similar total protein intakes, differences in protein source, amino acid composition, and digestion–absorption kinetics can influence protein utilisation and the expected clinical effect in critically ill patients (1, 31–33). High biological value proteins generally provide a complete essential amino acid profile and high digestibility, while leucine-rich proteins may more readily reach the threshold required to stimulate muscle protein synthesis (12, 31–33). Accordingly, the design of nutritional interventions should consider not only total protein delivery but also the rate and quality of amino acid provision.

Rapidly digested dairy proteins, particularly whey, are characterised by high leucine content and rapid amino acid availability and may therefore provide a stronger early anabolic stimulus under constrained energy delivery (31–33). More slowly digested proteins such as casein provide a more sustained amino acid release profile and may be relevant in continuous or recovery-oriented feeding scenarios (33). Plant-derived and egg-derived proteins may serve as alternatives when animal proteins are unsuitable, but phenotype-specific evidence in critical illness remains limited (34–39). Overall, current evidence does not support a clearly superior single protein source for all critically ill patients, and the choice of protein type should be individualised according to feeding pattern, metabolic tolerance, and clinical context (31–39).

### 3.3 Protein supplementation and immune function

Functional amino acids and high-quality protein sources contribute to the interaction between protein supplementation and immune function. Glutamine and arginine have been linked to lymphocyte proliferation, macrophage activity, cytokine regulation, and antioxidant defence, but their clinical effects appear to depend strongly on phenotype, timing, and organ function (29, 40–49). In particular, moderate glutamine supplementation has been associated with favourable signals in some earlier studies, whereas high-dose glutamine in critically ill patients with multiple organ failure has been linked to adverse outcomes (40–46, 49, 50).

Arginine may promote wound healing and immune recovery in selected trauma, surgical, and burn settings, but in typical septic shock populations its safety and net clinical benefit remain uncertain because of concerns regarding nitric oxide-mediated vasodilation and haemodynamic instability (29, 41, 47–49). Overall, adequate protein provision remains the primary priority, whereas functional amino acids should be used cautiously and in a phase-specific manner (29, 40–49).

## 4 Clinical evidence for protein supplementation

Current evidence supports appropriate protein provision as a core component of nutritional support in critically ill patients, but a simple dose–response relationship in which higher protein intake consistently improves outcomes has not been confirmed (2, 51–56). International guidelines generally recommend progressive rather than early aggressive escalation: protein intake in critically ill adults should usually reach at least 1.2 g/kg/day, with stepwise advancement toward approximately 1.3 g/kg/day during the acute phase (2, 51, 56). In severe burns, higher targets of 1.5–2.0 g/kg/day are more consistently recommended because of sustained hypermetabolism, exudative losses, and wound-healing demands (25, 57, 58).

In sepsis and general ICU populations, randomised trials and meta-analyses comparing higher versus standard protein delivery have yielded inconsistent conclusions. Although some studies suggest attenuation of muscle loss or improvement in intermediate nutritional markers, consistent benefits on mortality or other major clinical outcomes have not been demonstrated (52–55). These findings support progressive achievement of guideline targets rather than indiscriminate early escalation of protein delivery in septic critical illness (2, 51–56).

In contrast, evidence in severe burns more consistently supports higher protein requirements. Adult burn targets are typically set at 1.5–2.0 g/kg/day to counter prolonged hypermetabolism, ongoing protein losses, and wound repair needs (25, 57, 58). Across phenotypes, the route and timing of delivery remain as important as nominal dose: enteral-first strategies are preferred, and parenteral supplementation should be used selectively when enteral targets cannot be achieved (56, 59–61).

### 4.1 Controversy and gaps

The main uncertainty is when escalation during the early acute phase becomes beneficial rather than futile or harmful, particularly in patients with evolving organ dysfunction or poor metabolic tolerance. Future studies should define phenotype- and phase-specific escalation algorithms with prespecified safety thresholds.

## 5 Medical nutrition strategies and practical pathways for protein supplementation

Conceptually, phase-adapted protein-focused nutrition in critical illness can be organised along the ebb–flow–post-acute trajectory. In practice, this means an enteral-first strategy after haemodynamic stabilisation, stepwise advancement of protein delivery toward guideline-level targets, avoidance of excessive early metabolic load, and repeated reassessment of tolerance and target attainment (2, 16, 51, 56). Supplemental parenteral strategies should generally be reserved for situations in which enteral delivery remains insufficient after structured attempts to optimise feeding (56, 59–61).

Protein provision and energy delivery are related but not interchangeable targets. Earlier prioritisation of protein may support tissue repair and limit loss of fat-free mass, whereas energy delivery should be advanced more cautiously to reduce the risk of overfeeding and metabolic complications. Reassessment at 24–48 h and subsequent milestones should integrate achieved intake with metabolic tolerance and safety signals, including energy expenditure estimates, urea-related indices, glycaemic control, triglycerides, hepatobiliary function, ventilatory burden, and gastrointestinal tolerance (2, 56).

In sepsis and mixed ICU populations, current practice favours early but cautious initiation of enteral nutrition once haemodynamic resuscitation is largely completed and vasopressor requirements are stable or decreasing (2, 51, 56, 62–64). A pragmatic approach is to begin with modest protein delivery during the first 1–3 days and titrate toward approximately 1.2–1.3 g/kg/day by day 3, guided by circulatory stability, gastrointestinal tolerance, renal function, and nutrition risk (2, 51, 56, 65). Very high early doses do not consistently improve outcomes and may increase metabolic burden in patients with significant organ dysfunction (52–55, 65).

Patients with severe burns represent the opposite end of the spectrum, with extreme and prolonged hypermetabolism, marked protein oxidation, and continuous wound-related losses (3, 13–15, 66, 67). In this setting, protein requirements of 1.5–2.0 g/kg/day are commonly recommended, and early high-protein enteral nutrition is considered standard care when tolerated (3, 24, 25, 57, 58, 67). However, audits suggest that achieving prescribed targets remains difficult in routine practice (57, 58).

When enteral nutrition remains insufficient, parenteral nutrition becomes a selective adjunct rather than a primary modality. The EPaNIC trial and subsequent syntheses support delayed rather than very early parenteral supplementation in most critically ill adults (56, 59–61). In acute kidney injury without renal replacement therapy, protein escalation should remain cautious, whereas initiation of intermittent or continuous renal replacement therapy may justify re-titration toward higher targets because of extracorporeal amino acid losses and ongoing catabolism (2, 16, 23, 51, 56).

### 5.1 Controversies and knowledge gaps

Achieving protein targets remains limited by delivery failure, creating a persistent gap between prescribed goals and achieved bedside exposure. Future implementation trials should test pragmatic phase-adapted pathways, quantify delivered protein and energy, and evaluate feasibility and patient-centred outcomes alongside safety.

## 6 Targeted protein supplementation strategies: specific proteins and their derivatives

In nutritional interventions for critically ill patients, evaluating protein support solely by daily total protein intake is often insufficient to account for outcome heterogeneity across clinical phenotypes. Beyond quantity, differences in protein source, amino acid composition, digestion–absorption kinetics, and the incorporation of functional amino acid modules can differentially influence muscle protein turnover, wound healing, and protein synthesis in immune cells, which is particularly relevant in hypercatabolic populations such as sepsis and severe burns (1–3, 31–33, 40–48). Accordingly, this section synthesises the available evidence and key limitations for representative intact proteins and selected derivatives within overall protein strategies, with a focus on sepsis and severe burns (1–3, 31–33, 40–48, 115, 116). Beyond absolute protein dose, the temporal distribution of protein exposure may also influence myofibrillar protein synthesis and should be considered when translating mechanistic insights into critical care practice (125).

### 6.1 Whey protein

Whey protein is a high biological value protein source characterised by high leucine content and rapid digestion–absorption kinetics, leading to early amino acid availability and activation of anabolic signalling relevant to skeletal muscle protein synthesis (31, 32). This profile provides a physiological rationale for the use of whey-dominant formulations to support muscle protein accretion and limit negative nitrogen balance under conditions of accelerated proteolysis (31–33). In ICU practice, formulas with a relatively higher whey proportion may be considered when rapid amino acid availability is desirable, but phenotype-specific evidence in sepsis and severe burns remains limited and direct head-to-head trials are lacking (31–33, 68).

### 6.2 Casein

Casein is an intact bovine milk protein that forms a gastric clot and provides slower digestion–absorption kinetics with a more sustained amino acid release profile over time (33, 69). Compared with whey, casein is more closely associated with maintenance of positive whole-body protein balance over longer intervals rather than with a strong early anabolic peak (33). This profile may be relevant in continuous or recovery-oriented feeding scenarios, but direct evidence in sepsis and severe burns remains limited (33, 69).

### 6.3 Branched-chain amino acids (BCAAs)

Branched-chain amino acids, particularly leucine, are important modulators of skeletal muscle protein metabolism through mTOR-related signalling and may shift muscle protein turnover toward a more anabolic profile under catabolic stress (30, 70, 71). In sepsis and other hypercatabolic states, lower circulating BCAA concentrations have been associated with worse outcomes, suggesting potential value as metabolic or prognostic markers (12, 72). However, clinical evidence for BCAA-focused supplementation remains limited, and current data do not establish that intensified BCAA strategies improve mortality or organ function beyond achievement of total protein targets (70–74, 117).

### 6.4 Glutamine

Glutamine is widely regarded as a conditionally essential amino acid during critical illness, acting as a key substrate for enterocytes and immune cells, supporting interorgan nitrogen trafficking, and contributing to antioxidant defence via glutathione synthesis (40, 43–46). However, mechanistic plausibility and signals from earlier small trials or heterogeneous meta-analyses are insufficient to support routine supplementation; the clinical net benefit must be defined by adequately powered randomised controlled trials using patient-centred outcomes (43–46).

In severe burns, a randomised trial of glutamine supplementation did not establish clear clinically meaningful benefit sufficient to support routine supplementation (75). In critically ill adults more broadly, systematic reviews and meta-analyses have reported inconsistent efficacy and ongoing safety concerns, particularly in patients with severe organ dysfunction (43–46). Taken together, current evidence does not support routine glutamine supplementation in sepsis-related critical illness. Any use should be highly selective, with careful attention to renal function, organ failure status, and protocolised monitoring (40, 43–46, 75, 118).

### 6.5 Arginine

Arginine is a constituent amino acid of proteins and a precursor for nitric oxide, polyamines, and creatine, thereby influencing microcirculatory perfusion, tissue repair, and immune cell function (29, 41, 47). In clinical nutrition, arginine-enriched immunonutrition—supported mainly by studies in surgical and trauma populations—has been associated with improved wound-related and infectious outcomes in selected settings, although mortality benefit has not been consistently demonstrated (41, 49).

In infectious shock and typical sepsis phenotypes, the safety of arginine remains controversial because increased substrate availability may augment nitric oxide-mediated vasodilation during systemic inflammation and haemodynamic instability (29, 47, 48). Accordingly, arginine enrichment is better reserved for patients with severe burns or major surgery when haemodynamics are stable and reparative demands are high, and it should not be used as a first-line protein-intensification strategy in early sepsis or during ongoing circulatory instability (29, 41, 47–49). Overall, phenotype-specific evidence in sepsis remains limited, and use should be guided by strict haemodynamic and metabolic monitoring with clear stop criteria.

### 6.6 Other amino acid supplementation

Beyond the functional amino acids described above, other amino acids may also have theoretical roles in critical illness. However, in the current reference list of this manuscript, there are no dedicated clinical studies directly supporting independent recommendations for methionine or threonine supplementation in sepsis or severe burns. Accordingly, this subsection should remain cautious in scope: at present, a more feasible approach is to ensure adequate provision indirectly by prioritising intact protein sources or enteral formulas with balanced amino acid patterns once total protein targets are achieved. High-dose single–amino-acid intensification should not be incorporated into routine care and should remain confined to mechanistic research or exploratory clinical trials with predefined safety monitoring.

#### 6.6.1 Controversy and gaps

Across protein sources and amino-acid derivatives, apparent advantages are frequently inferred from mechanistic plausibility, whereas clinical benefits remain inconsistent and likely vary by phenotype, dose, and phase window. Safety boundaries are particularly consequential when indirect harms or adverse signals have been reported, necessitating cautious interpretation, explicit exclusion criteria, and protocolised monitoring. Future trials should prioritise responder stratification and head-to-head comparisons against iso-nitrogenous controls within prespecified timing windows.

## 7 Modulating protein metabolism with micronutrients

In nutritional support for critically ill patients, micronutrients play an important modulatory role in the efficiency of protein utilisation, tissue repair, and the metabolic response to stress. Zinc, selenium, and vitamin C are involved in redox homeostasis, wound healing, and the broader biochemical environment required for effective protein use in critical illness (76–83). In parallel, probiotics and related gut microbiota-directed interventions may indirectly influence protein utilisation by modifying the intestinal environment and nutrient handling, thereby creating more favourable conditions for absorption and maintenance of lean tissues (84–86). In addition, n-3 polyunsaturated fatty acids and selected functional amino acids have been investigated as metabolic modulators that may affect inflammation, microcirculation, and the efficiency of protein use (75, 87–90, 119). Re-examining these modulatory factors from the perspective of protein supplementation strategies helps refine the framework of protein-targeted nutritional interventions in critical illness.

### 7.1 Micronutrients with direct effects on protein metabolism

Zinc and selenium are key micronutrients involved in protein synthesis, antioxidant defence, and tissue repair. In critical illness, deficiency of either trace element may impair recovery, while correction of deficiency is a reasonable component of nutritional support (80–83). In severe burns, trace element supplementation has been associated with higher tissue concentrations and lower local protein degradation rates (77, 91).

Vitamin C is closely linked to collagen synthesis, connective tissue stability, and oxidative stress responses. In burns and surgical critical illness, vitamin C-containing antioxidant strategies have mainly been studied in relation to capillary permeability, oxidative stress, fluid requirements, and tissue repair (76–79). Taken together, micronutrient deficiencies should be systematically assessed and corrected to avoid situations in which higher protein provision is accompanied by impaired synthetic efficiency or delayed tissue recovery (76–83, 120). Micronutrient therapy should therefore be individualised according to deficiency risk, oxidative stress burden, feeding route, and the broader metabolic context of critical illness (124). Thiamine deficiency is also relevant in sepsis and critical illness, but adjunctive thiamine should be regarded as a phenotype-specific metabolic support strategy rather than a routine protein-anabolic intervention (127, 128).

### 7.2 Use of probiotics to modulate protein metabolism

The gastrointestinal tract is central to protein digestion, amino acid absorption, and integration of metabolic signals. In critical illness, probiotics and synbiotics have been investigated mainly in relation to intestinal barrier function, infection-related outcomes, and the intestinal environment rather than as direct anabolic therapies (84–86). From the perspective of protein metabolism, their relevance lies in the possibility that a more stable intestinal milieu may support nutrient utilisation and maintenance of lean tissues.

Current evidence suggests that, when strain selection is rigorous and safety monitoring is robust, probiotics and synbiotics may exert indirect beneficial effects on nutrient handling by improving the intestinal environment and limiting harmful metabolic disturbances (84–86, 92). However, safety concerns remain relevant in patients with profound immunosuppression or severe barrier disruption, and routine use in critical illness is not supported (121).

### 7.3 Use of metabolic therapies to modulate protein metabolism

Metabolic therapies may influence the balance between protein synthesis and degradation by modulating fatty acid utilisation, inflammatory signalling, and cellular energy status. n-3 polyunsaturated fatty acids, particularly EPA and DHA, have been studied in critically ill patients and burn populations mainly for their effects on inflammation and organ function rather than for direct anabolic outcomes (87–90, 122). Available evidence suggests that fish-oil-containing enteral or parenteral formulations may modify the metabolic milieu for protein utilisation, but direct evidence for improvements in muscle mass and functional recovery remains limited (87–90).

Among functional amino acids, glutamine has been more directly evaluated in the current reference set. In severe burns, randomised data did not establish sufficient clinical benefit to support routine glutamine supplementation (75). Taken together, metabolic interventions should be regarded as adjunctive modulators of the environment for protein utilisation rather than substitutes for adequate protein delivery, and their use should remain phenotype-specific and guided by organ function and metabolic tolerance.

## 8 Grey areas and controversies: challenges and uncertainties in protein supplementation

Building on the matrix presented in Table 1, this section focuses on current grey areas and unresolved controversies related to protein-focused nutritional strategies in critical illness. Although protein supplementation is widely recognised as a central component of nutritional therapy in critically ill patients, significant gaps and inconsistencies remain in the evidence base regarding its optimal implementation. Key uncertainties include the ideal protein dose, timing of initiation, and the most suitable protein sources for different clinical phenotypes, such as sepsis or severe burns. ASPEN- and ESPEN-related guidance generally proposes intake ranges around 1.2–2.0 g/kg/day or stepwise escalation toward approximately 1.3 g/kg/day, yet practical recommendations remain insufficiently stratified across disease phenotypes and phases of illness, and evidence certainty for many recommendations remains limited (2, 16, 51, 56, 115). In clinical practice, protein prescriptions therefore often fluctuate between guideline targets and the patient’s actual tolerance (2, 51, 56, 92, 123). Glutamine is a representative example in which strong mechanistic rationale has not translated into consistent clinical benefit: randomised trials in severe burns and mixed ICU cohorts failed to improve outcomes and, in some settings, raised safety concerns; therefore, routine use remains unjustified (8, 43–46, 75).

**Table 1:** Phenotype-informed metabolic and protein supplementation strategies across critical illness contexts (sepsis vs. severe burns) and recovery.

Modulating factors/interventions	Severe burn	Sepsis/critical illness	ICU survivor/rehabilitation
Early protein-enriched EN	R: Initiate EN within 24 h; target protein delivery 1.5–2.0 g/kg/day when tolerated (25, 57)	R: Initiate EN within 24–48 h after resuscitation and stabilisation, with stepwise escalation of protein delivery toward guideline-level targets (2, 51, 56, 63)	R: Maintain adequate protein intake during recovery, often at least approximately 1.2 g/kg/day when tolerated, with high-protein diet and/or oral nutritional supplements (92, 94, 95)
Glutamine	NR: A randomised trial in severe burns did not demonstrate sufficient clinical benefit to support routine glutamine supplementation (75)	NR: High-dose glutamine has not shown consistent benefit in general critical illness and may be harmful in selected patients; routine use is not recommended (43–46, 50)	TBE: Direct evidence for routine use during recovery is lacking.
Arginine / arginine-enriched immunonutrition	OI: Short-term immunonutrition may be considered in selected surgical/trauma settings, but direct evidence for major burns remains limited (29, 41, 49)	NR: Arginine-enriched immunonutrition is not recommended in severe sepsis because haemodynamic safety remains uncertain (29, 47, 48)	TBE: Evidence in rehabilitation settings remains limited.
n-3 polyunsaturated fatty acids (fish oil-enhanced EN/PN)	OI: In major burns, fish oil-derived omega-3 supplementation has been evaluated mainly in small trials and meta-analyses; reported benefits are inconsistent (89, 90)	OI: Results of *ω*-3-enriched formulas are inconsistent, and benefits on mortality and muscle outcomes remain uncertain; fish-oil-containing lipid emulsions may be considered when PN is indicated (87, 88, 103)	TBE: Evidence for benefits on long-term muscle mass and function remains insufficient.
Vitamin D repletion	R: Vitamin D deficiency is common in critical illness and should be assessed and corrected using standard repletion protocols (104–106)	R: Guidelines support testing and correction of significant deficiency, although mortality benefit has not been consistently demonstrated (104–106)	R: An important component of rehabilitation nutrition, particularly when deficiency is present (104–106)
High-dose IV vitamin C	OI: Small studies in burns suggest reduced fluid resuscitation requirements, but effects on protein metabolism and outcomes remain unclear (76, 77)	OI: Evidence in sepsis and critical illness remains inconsistent; routine use is not recommended outside clinical trials (76–79)	TBE: Lack of evidence for improving muscle or functional outcomes during long-term rehabilitation
Selenium supplementation	S: Deficiency can be corrected with regular doses; routine high-dose intravenous bolus is not recommended (77, 80, 81, 91)	OI: High-dose sodium selenite has not improved prognosis consistently and is not recommended routinely; supplement when deficiency is documented (80, 81)	TBE: There is no basis for routine enhanced supplementation during the recovery period
Probiotics / synbiotics	TBE: Evidence in burn populations remains very limited; potential benefits on infections and gut barrier function require confirmation (84–86)	TBE: In critical illness, probiotics/synbiotics may improve the intestinal environment, but routine use is not supported because efficacy and safety remain uncertain (84–86)	OI: Potential benefits after discharge remain exploratory and require further follow-up data.
β-blockade (propranolol in burns; esmolol in septic shock)	R: Randomised trials have shown that propranolol reduces hypermetabolism and muscle protein breakdown and improves lean body mass in severe burns (67, 107, 108)	OI: In sepsis, *β*-blockers are used mainly for haemodynamic management, whereas their relationship to protein metabolism remains uncertain.	TBE: Evidence for use during recovery to preserve muscle mass remains limited.
Intensive insulin therapy	NR: Intensive glycaemic control increases hypoglycaemia and may increase mortality; it is not recommended as a protein-protection strategy (109–112)	NR: Current sepsis guidance supports moderate glucose control rather than intensive treatment (109–112)	NR: Intensive blood glucose lowering during the recovery period is unlikely to improve protein metabolism and may increase hypoglycaemia risk (109–112)
L-carnitine and related preparations	TBE: Evidence in burn populations is limited and largely derived from older controlled nutrition models; no consistent improvement in clinically relevant outcomes has been demonstrated (113)	OI: In septic shock, adjunctive L-carnitine has not shown a consistent benefit on mortality or other hard endpoints in randomised trials (114)	TBE: Evidence for long-term follow-up and use during the recovery period is lacking

This table is structured as a context-based decision matrix. Severe burns and sepsis/medical critical illness are presented as distinct clinical phenotypes rather than chronological phases of a single disease. The ICU survivor/rehabilitation column represents the post-ICU stage that may apply across underlying diagnoses. Recommendation grades: Recommended (R), Suggested (S), Open to individualisation (OI), Not recommended (NR), and To be explored (TBE), assigned according to the overall balance of evidence and guideline consensus. Abbreviations: enteral nutrition (EN), parenteral nutrition (PN), intravenous (IV), randomised controlled trial (RCT), grams per kilogram per day (g/kg/day). Protein dose targets and adjunctive interventions should be interpreted in conjunction with the evolving clinical trajectory, including haemodynamic stability, gastrointestinal tolerance, and renal and hepatic function, and should be reassessed iteratively during ICU care.

### 8.1 Challenges of individualisation: balancing dose and tolerance across metabolic phenotypes

In patients with sepsis and severe burns, protein requirements are highly heterogeneous. Sustained catabolism, rapid skeletal muscle loss, and wound-healing demands often justify higher protein targets, particularly in major burns and selected high-risk critically ill populations (2, 3, 51, 56, 67, 124). However, renal dysfunction, hepatic dysfunction, and haemodynamic instability constrain the safe implementation of higher protein doses, and randomised trials suggest that early aggressive escalation does not consistently improve outcomes and may even be harmful in selected patients with poor metabolic tolerance or ongoing organ dysfunction (2, 16, 51–56, 93, 119). Conversely, in later phases of illness or rehabilitation, achieving at least guideline-level protein delivery appears more favourable for preservation of lean mass and recovery-related outcomes (92, 94–96). This highlights the need for phenotype-stratified strategies that integrate metabolic state, organ function, and nutrition risk.

### 8.2 Dose, timing, and protein source: inconsistent evidence and uncertainty about optimal windows

Randomised trials and meta-analyses have yielded divergent conclusions regarding the optimal dose and timing of protein supplementation (125). Overall, the current evidence suggests that matching dose to the temporal window and phase of illness may be more important than any single absolute dose threshold (52–55, 92–95, 118). In parallel, uncertainty remains regarding the clinical value of specific protein sources and functional amino acid-enriched formulations. Although mechanistic differences among intact proteins and amino acid modules are biologically plausible, existing studies are small, heterogeneous, and difficult to compare, and no clearly superior protein type has been established for sepsis or severe burns (31–37, 40–49, 70–75).

### 8.3 Barriers to implementation: the gap between theoretical targets and bedside achievement

Even when protein targets are defined on the basis of guidelines and clinical studies, achieving them at the bedside remains difficult because gastrointestinal dysfunction, haemodynamic instability, feeding intolerance, and frequent interruptions due to procedures or investigations often limit advancement of enteral nutrition (63, 97–100, 132–135). Attempts to compensate for insufficient enteral delivery with parenteral nutrition may also increase infectious and metabolic risk when introduced too early or inappropriately (56, 59–61, 136, 143). In addition, traditional biochemical markers such as albumin and prealbumin are strongly influenced by inflammation and fluid shifts and therefore do not reliably reflect whether protein intake is adequate or effectively utilised (20, 21). As a result, achieved protein delivery often falls short of prescribed targets, highlighting the need for more practical feedback systems integrating indirect calorimetry, serial muscle assessment, and systematic documentation of feeding interruptions (96, 98–102, 126). In selected overweight critically ill patients, use of a very-high-intact-protein enteral formula may improve achievement of protein targets, although generalisability remains limited (123). Fiber-containing enteral formulas may improve feeding tolerance and gastrointestinal outcomes in selected critically ill patients, although the evidence remains heterogeneous and does not justify universal routine use (129).

## 9 Future research directions

Using the same matrix framework as a conceptual background, the following section outlines priority areas for future research on protein supplementation in sepsis and severe burns. Given the persistent uncertainties in the current evidence base, research on protein-focused nutritional interventions in critical illness is gradually shifting from simply increasing total intake toward phenotype- and phase-based precision supplementation. Future work needs to progress in parallel across three domains: optimisation of supplementation regimens, multi-omics driven individualised strategies, and evaluation of long-term outcomes.

### 9.1 Optimising protein supplementation based on disease phenotypes

Future randomised controlled trials should re-examine protein dose and timing within a more granular framework of disease stratification rather than treating ICU patients as a relatively homogeneous population. Sepsis, severe burns, and postoperative critical illness differ substantially in metabolic stress, muscle loss, and vulnerability of organ dysfunction, and future trials should therefore compare standard versus higher protein intakes and early versus delayed initiation strategies across distinct phases of illness, with endpoints including mortality, organ function, skeletal muscle outcomes, and functional recovery (52–55, 67, 92, 94, 95, 127). Implementation-related variables such as actual target attainment rates, feeding interruptions, and the balance between enteral and parenteral delivery should also be incorporated as prespecified metrics so that findings more closely reflect real-world practice (92).

### 9.2 Multi-omics and precision nutrition: constructing protein requirement phenotypes

With advances in metabolomics, proteomics, and immune phenotyping, divergent responses to the same nutritional intervention may be investigated more systematically. Future work should integrate body composition tools such as muscle ultrasound and CT-based quantitative measures of muscle mass with inflammatory and metabolic variables in order to develop risk scores or decision models that predict individual responses to higher versus standard protein strategies (19, 96). Such phenotype-guided frameworks may help define more precise protein targets and timing strategies in different subgroups of critically ill patients (128). This precision-oriented direction is consistent with recent calls for personalised nutrition therapy in critical care and sepsis (126).

### 9.3 Long-term functional outcomes and recovery-phase protein strategies

At present, most studies focus primarily on ICU or in-hospital outcomes, whereas evidence linking protein delivery to long-term recovery after discharge, including muscle preservation, exercise capacity, and quality of life, remains limited (92, 94–96). Existing cohort and observational data suggest that nutrition adequacy, muscle mass, and protein delivery are associated with clinically relevant outcomes, but whether higher protein intake consistently improves longer-term survival or functional recovery remains uncertain (92, 94–96, 129). Future research should therefore extend observation windows beyond hospital discharge and jointly evaluate lean body mass, muscle strength, physical function, and activities of daily living.

## 10 Conclusion

Protein metabolic derangement is a hallmark of critical illness and contributes substantially to adverse prognosis and impaired recovery. Using sepsis and severe burns as representative hypercatabolic phenotypes, this review summarises current evidence on altered protein metabolism, assessment of protein requirements, and supplementation strategies. Overall, appropriately timed and dosed protein supplementation may attenuate hypercatabolism, preserve lean body mass, and support wound healing and organ recovery, but its benefit depends not only on total intake but also on illness phase, disease phenotype, organ function, and metabolic tolerance.

Current evidence supports a shift from a high-dose-centred approach toward phenotype-oriented and dynamically adjusted protein-focused nutrition. In practice, early enteral nutrition after relative haemodynamic stabilisation, with progressive titration of protein intake to at least 1.2–1.3 g/kg/day and cautious escalation in selected hypercatabolic phenotypes such as severe burns, appears to be a reasonable strategy. These principles are consistent with both the ESPEN 2019 ICU guideline and the ESPEN 2023 practical update (2, 56).

Important uncertainties remain regarding optimal dose and timing across disease phenotypes and illness phases, particularly during the early unstable stage of critical illness. Evidence for specific protein sources and functional amino acids also remains limited, and current monitoring tools are insufficient for precise individualisation. Future studies should therefore develop phenotype-specific protein strategies integrating metabolic, imaging, functional, and long-term patient-centred outcomes.
